# Dysbiotic human oral microbiota alters systemic metabolism via modulation of gut microbiota in germ-free mice

**DOI:** 10.1080/20002297.2022.2110194

**Published:** 2022-08-11

**Authors:** Kyoko Yamazaki, Eiji Miyauchi, Tamotsu Kato, Keisuke Sato, Wataru Suda, Takahiro Tsuzuno, Miki Yamada-Hara, Nobuo Sasaki, Hiroshi Ohno, Kazuhisa Yamazaki

**Affiliations:** a Division of Periodontology, Niigata University Graduate School of Medical and Dental Sciences, Niigata Japan; b Laboratory for Intestinal Ecosystem, RIKEN Centre for Integrative Medical Sciences (IMS), Kanagawa Japan; c Laboratory of Mucosal Ecosystem Design, Institute for Molecular and Cellular Regulation, Gunma University, Maebashi, Gunma Japan; d Immunobiology Laboratory, Graduate School of Medical Life Science, Yokohama City University, Kanagawa Japan; e Laboratory for Microbiome Sciences, RIKEN Center for Integrative Medical Sciences, Yokohama, Japan

**Keywords:** Oral, gut, microbiome, liver, transcriptome, metabolome

## Abstract

**Background:**

The effect of oral microbiota on the intestinal microbiota has garnered growing attention as a mechanism linking periodontal diseases to systemic diseases. However, the salivary microbiota is diverse and comprises numerous bacteria with a largely similar composition in healthy individuals and periodontitis patients.

**Aim:**

We explored how health-associated and periodontitis-associated salivary microbiota differently colonized the intestine and their subsequent systemic effects.

**Methods:**

The salivary microbiota was collected from a healthy individual and a periodontitis patient and gavaged into C57BL/6NJcl[GF] mice. Gut microbial communities, hepatic gene expression profiles, and serum metabolites were analyzed.

**Results:**

The gut microbial composition was significantly different between periodontitis-associated microbiota-administered (PAO) and health-associated oral microbiota-administered (HAO) mice. The hepatic gene expression profile demonstrated a distinct pattern between the two groups, with higher expression of lipid and glucose metabolism-related genes. Disease-associated metabolites such as 2-hydroxyisobutyric acid and hydroxybenzoic acid were elevated in PAO mice. These metabolites were significantly correlated with characteristic gut microbial taxa in PAO mice. Conversely, health-associated oral microbiota were associated with higher levels of beneficial serum metabolites in HAO mice.

**Conclusion:**

The multi-omics approach used in this study revealed that periodontitis-associated oral microbiota is associated with the induction of disease phenotype when they colonized the gut of germ-free mice.

## Introduction

The oral cavity and lower gastrointestinal tract are anatomically connected, and most symbiotic microbial communities in humans inhabit these regions. The saliva contains a significant number of oral microbiota that are constantly swallowed into the gastrointestinal tract. However, the effect of the oral microbiota on the gut microbiota and the subsequent negative impact on systemic health has generally been disregarded, except in the case of cirrhosis [1], inflammatory bowel disease [2], and colorectal cancer [3]. Furthermore, because there is a barrier between the oral cavity and intestinal tract, such as gastric acid and bile acid, most swallowed oral microbiota are considered to be killed and never reach the intestinal tract. However, it is increasingly evident that a significant amount of the oral microbiota flow into the gut and are members of the gut microbiota in systemically healthy individuals [4]. Thus, unhealthy oral microbiota, for example, those observed in periodontitis patients, may distinctly affect the gut microbiota and subsequent systemic conditions.

Studies analyzing the salivary microbiota in periodontitis patients have reported that the composition of the microbiota reflects changes in the subgingival microbiota of the lesions. The changes include an increase in the proportion of periodontopathic microbiota, mainly *Porphyromonas gingivalis*, and a decrease in the proportion of microbial groups associated with healthy periodontal tissues [5]. We previously showed that oral gavage of *P. gingivalis*, a representative periodontopathic bacterium, induced changes in the gut microbiota composition, metabolic changes, and disease-associated pathologies that differ from those of *Prevotella intermedia*, another periodontopathic bacterium with weaker pathogenicity [6–8]. These results have drawn attention to the role of the oral microbiota in the gut ecology in the periodontitis-systemic disease connection. However, periodontal health-associated microbiota and disease-associated microbiota show distinct colonization patterns in germ-free (GF) mice, suggesting cooperative and competitive interactions among the complex oral microbiota for gut colonization [9]. This suggests that the oral microbial community in healthy individuals and periodontitis patients may have a more complex effect on the gut microbial community and subsequent systemic conditions.

Therefore, in this study, we administered salivary microbiota from a periodontitis patient and a periodontally healthy individual to GF mice and analyzed the colonization in the gut, hepatic gene expression profile, and serum metabolites. Our study further highlights the role of periodontitis in systemic health by affecting the gut ecology.

## Materials and methods

### Collection and analysis of saliva samples

Saliva samples from a periodontitis patient and a periodontally healthy individual, both of whom were otherwise systemically healthy and had used no antimicrobials within the past 6 months, were selected based on the clinical parameters and bacterial composition determined using our ongoing study’s cohort data. The experimental protocol was approved by the Institutional Review Board of the Niigata University Medical and Dental Hospital (approval number 2015–5098), and registered with the University Hospital Medical Information Network – Clinical Trials Registry (UMIN-CTR) (Registration number UMIN000036801). Informed consent was obtained from all the participants.

Unstimulated saliva was collected at least 2 h after breakfast (no food and drink during the interval) and immediately frozen at – 80°C. Frozen saliva was thawed and immediately suspended in an equal volume (w/v) of phosphate-buffered saline (PBS) containing 20% glycerol/PBS. The samples were then centrifuged at 12,000 rpm for 15 min at 20°C and the precipitates were suspended in 100 μL of PBS. The isolation of microbial DNA, 16S rRNA sequencing, and data analysis from human saliva samples were performed as described [10]. The demographic data of the donors and their microbial compositions demonstrated characteristic features of healthy periodontium and periodontitis, respectively (S1 Table and S2 Table).

## Generation of human oral microbiota-associated mice and sample collection

Five-week-old male C57BL/6NJcl[GF] mice generated at the Technical Service Department of CLEA Japan, Inc. (Fujinomiya, Japan) were acclimatized for one week prior to the commencement of the experiment in vinyl isolators under germ-free conditions. The mice were fed sterile regular chow. This study was approved by the Institutional Animal Care and

Use Committee of Niigata University (permit number; SA00529). All experiments were performed in accordance with the Regulations and Guidelines on Scientific and Ethical Care and Use of Laboratory Animals of the Science Council of Japan, enforced on 1 June 2006 and ARRIVE guidelines.

The mice were fed regular sterile food and, after acclimatization, were inoculated with 100 µL of oral microbiota samples via oral gavage (disposable feeding needle; Kenis Ltd., Osaka, Japan).

The liver, epididymal adipose tissue, and blood were obtained 6 weeks after the oral gavage [11].

## Histological analyses

Part of the left and medial lobes and epididymal were fixed in neutral-buffered formalin. After deparaffinization and rehydration, paraffin-embedded sections (5 µm in thickness) were stained with hematoxylin and eosin (H&E). For macrophage staining for epididymal adipose tissue, the sections were stained with rat anti-mouse F4/80 antibody (ABd Serotec, Raleigh, NC; 1:50 dilution). The stained sections were visualized by fluorescence microscopy (Biozero BZ-X710; Keyence Corporation, Osaka, Japan).

## DNA extraction and *16S rRNA* gene sequencing of fecal samples

DNA extraction from the feces and gut microbiota profiling were carried out as described previously [8].

The V4 variable region (515 F-806 R) of the samples was sequenced using Illumina MiSeq, according to the method described by Kozich et al. [12]. Primers with adaptor sequences for the Illumina MiSeq platform were as follows: forward primer, 5ʹ-AATGATACGGCGACCACCGAGATCTACAC NNNNNNNN TATGGTAATTGTGTGCCAGCMGCCGCGGTAA-3ʹ; and reverse primer, 5ʹ-CAAGCAGAAGACGGCATACGAGAT NNNNNNNN AGTCAGTCAGCCGGACTACHVGGGTWTCTAAT-3ʹ. The ‘NNNNNNNN’ sequence unique to each sample was attached to the primer for multiplexing. The PCR mixture contained 15 pmol of each primer, 50 ng microbial DNA, 4 µL of 2 mM dNTP mixture, 5 µL 10 × Ex Taq buffer, 0.25 µL Ex Taq Hot Start Version (Takara Bio Inc., Shiga, Japan), and sterile water to reach a final volume of 50 µL.

The PCR conditions were as follows: 95°C for 2 min, followed by 25 cycles of 95°C for 30s, 55°C for 15s, 72°C for 1 min, and 72°C for 3 min. The PCR products were purified using AMPure XP (Beckman Coulter, Brea, CA) and quantified using a Quant-iT PicoGreen dsDNA Assay Kit (Life Technologies Japan, Tokyo, Japan). Mixed samples were prepared by pooling approximately equal amounts of the PCR amplicons from each sample. The pooled library was analyzed using a TapeStation High-Sensitivity DNA 1000 assay (Agilent Technologies, Santa Clara, CA). Real-time PCR quantification of the pooled library was performed using an NEBNext Library Quant Kit for Illumina (New England Biolabs Japan Inc., Tokyo, Japan) according to the manufacturer’s instructions.

Based on quantification, the sample library was denatured and diluted. A DNA library containing 20% denatured PhiX spike-in was sequenced by MiSeq using a 500 cycles kit (Illumina Inc., San Diego, CA).

Amplicon sequence variants (ASVs) were inferred from denoised reads using DADA2 [13] (v.1.18.0). ASV taxonomy was assigned based on a comparison with the SILVA database v138 [14]. β-Diversity was calculated using weighted and unweighted UniFrac distances based on the ASV distribution across samples and visualized using principal coordinate analysis (PCoA). Permutational multivariate analysis of variance of UniFrac distances was performed using the adonis function in the vegan package (v2.5.7) with 1,000 permutations. Differentially abundant taxa at the genus level were determined using the ANCOM-BC package (v.1.0.5) [15].

## Metabolome analysis

Twenty-five microliters of serum were added to 100 µL of methanol containing internal standards (H3304-1002, Human Metabolome Technologies, Inc. (HMT), Tsuruoka, Yamagata, Japan) at 0°C to suppress enzymatic activity. The extract solution was thoroughly mixed with 75 µL of Milli-Q water, then 150 µL of the mixture was centrifugally filtered through a Millipore 5-kDa cutoff filter (ULTRAFREE MC PLHCC, HMT) at 9,100 × *g* and 4°C for 120 min to remove macromolecules. The filtrate was then evaporated to dryness in a vacuum and reconstituted in 25 µL of Milli-Q water for metabolomic analysis at HMT.

Metabolome analysis was conducted according to HMT’s *Basic Scan* package, using capillary electrophoresis time-of-flight mass spectrometry (CE-TOFMS) based on previously described methods [16,17]. CE-TOFMS analysis was conducted using an Agilent CE capillary electrophoresis system equipped with an Agilent 6210 time-of-flight mass spectrometer (Agilent Technologies, Inc., Santa Clara, CA). The systems were controlled by the Agilent G2201AA ChemStation software version B.03.01 (Agilent Technologies) and connected by a fused silica capillary (50μm *i.d*.×80cm total length) with a commercial electrophoresis buffer (H3301-1001 and I3302-1023 for cation and anion analyses, respectively, HMT) as the electrolyte. The spectrometer was scanned from *m/z* 50 to 1,000, and the peaks were extracted using the MasterHands automatic integration software (Keio University, Tsuruoka, Yamagata, Japan) to obtain peak information, including *m/z*, peak area, and migration time (MT) [18]. Signal peaks corresponding to isotopomers, adduct ions, and other product ions of known metabolites were excluded, and the remaining peaks were annotated according to the HMT metabolite database, based on their *m*/*z* values and MTs. Subsequently, the areas of the annotated peaks were normalized to the internal standards and sample amounts to obtain the relative levels of each metabolite. We absolutely quantified 277 primary metabolites based on one-point calibrations using their respective standard compounds. Hierarchical cluster analysis and principal component analysis (PCA) [19] were performed using proprietary MATLAB and R programs, respectively. Detected metabolites were plotted on metabolic pathway maps using the VANTED software [20].

## DNA microarray analysis

Total RNA from the tissue samples was extracted using TRIzol reagent (Molecular Research Center) 24 h after the final microbial or sham administration and quantified using a NanoDrop 2000 (Thermo Scientific, Wilmington, DE). Total RNA was labeled and hybridized to an Agilent SurePrint G3 Mouse Gene Expression 8 × 60 K mRNA microarray chip (Agilent Technologies). All microarray experiments were conducted by Macrogen, Japan (Kyoto, Japan).

Microarray results were extracted using the Agilent Feature Extraction software v11.0 (Agilent Technologies). Hierarchical cluster analysis was performed using complete linkage and Euclidean distance as similarity measures. PCoA plot with Bray-Curtis dissimilarity was constructed using the vegan package and visualized using ggplot2. Gene enrichment and functional annotation analyses for the significant probe list were performed using Gene Ontology (GO) (www.geneontology.org/). All data analyses and visualization of differentially expressed genes were conducted using R 3.3.2 (www.r-project.org).

## Quantitative real-time polymerase chain reaction (qPCR)

Total RNA was isolated using the TRI Reagent® (Molecular Research Center, Inc., Cincinnati, OH), according to the manufacturer’s instructions. cDNA was synthesized using Transcriptor Universal cDNA Master (Roche Molecular Systems, Pleasanton, CA). FastStart Essential DNA Green Master (Roche Molecular Systems) was used to amplify the mRNA in a LightCycler® 96 (Roche Molecular Systems) for qPCR analysis. The expression of each mRNA was normalized to that of GAPDH, using the ^ΔΔ^Ct method. TaqMan Gene Expression Assays (Life Technologies Japan, Ltd., Tokyo, Japan) were used for *Neat1* and *Acta2* quantification. The other oligonucleotide sequences (Thermo Fisher Scientific, Waltham, MA) used in this study are listed in S3 Table.

## Endotoxin assay

Serum endotoxin levels were determined using the Limulus Amoebocyte Lysate Test (Toxicolor™ LS50M, Seikagaku Co., Tokyo, Japan), according to the manufacturer’s instructions. Serum samples were 1:4 diluted for the assay. Optical densities were measured using an enzyme-linked immunosorbent assay plate reader (Emax Plus; Molecular Devices, San Jose, CA) at 545 nm.

## Glucose tolerance tests

Glucose tolerance test was performed in order to analyze the effect of gut dysbiosis induced by oral pathobionts on glucose tolerance. After overnight fasting at the end of the experimental period, the mice were injected intraperitoneally with a single dose of 1 g of glucose per kilogram of body weight. Blood samples were collected through the tail vein before glucose injection and at 15, 30, 60, 90, and 120 min after the injection. Blood glucose concentrations were immediately determined using a glucose pilot assay (Aventir Biotech, LLC, CA).

## Other statistical analyses

The correlation between microbial taxa and metabolites was evaluated using Spearman’s correlation coefficient test. Differences between the two groups were analyzed using Welch’s t-test (metabolome) or Mann–Whitney U-test. Statistical analyses were performed using GraphPad Prism version 9 (GraphPad Software, Inc., La Jolla, CA) and R software (version 4.0.3). All data were expressed as mean ± standard error of the mean. Statistical significance was set at *P* < 0.05.

## Results

### Periodontitis-associated oral microbiota induces phenotypic changes in liver and epididymal adipose tissue

**Figure 1.**
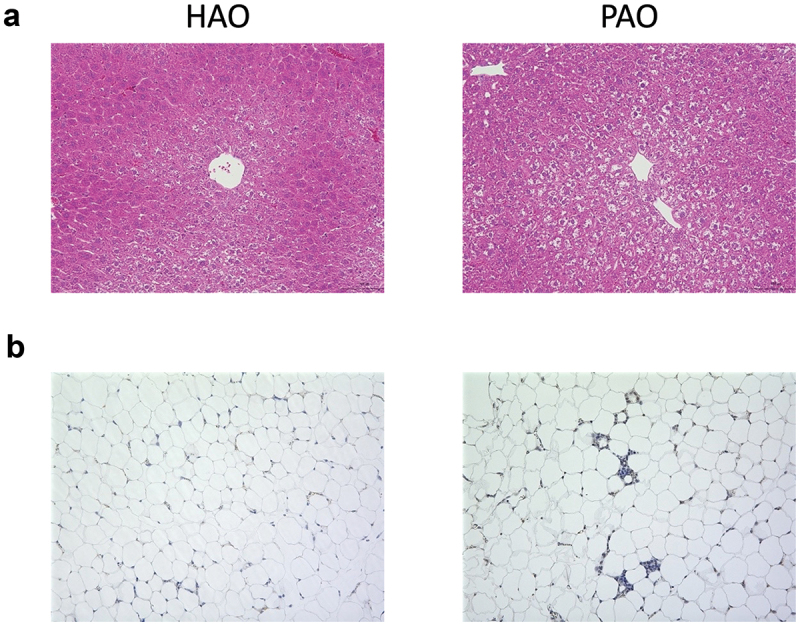
Oral gavage of periodontitis-associated oral microbiota and health-associated oral microbiota have distinct effects on liver and adipose tissue histology. (**A**) Hematoxylin and eosin staining of the liver (scale bars, 100 μm) of periodontitis-associated oral microbiota-administered (PAO) and health-associated oral microbiota-administered (HAO) mice. (**B**) Epididymal adipose tissues of PAO and HAO mice stained with a rat anti-mouse F4/80 primary antibody. Representative images (HAO #1 and PAO #1, respectively) of three HAO and four PAO mice.

Periodontitis-associated oral microbiota-administered (PAO) mice exhibited hepatic steatosis, a sign of non-alcoholic fatty liver disease (NAFLD). However, health-associated oral microbiota-administered (HAO) mice demonstrated normal histology or much less steatosis. Furthermore, the number of macrophages infiltrating the adipose tissue tended to be higher in PAO mice than those in HAO mice (Figure 1 and S1 Figure D). In addition, glucose tolerance was diminished in PAO mice compared to HAO mice, despite no differences in the body and liver weight changes during the experimental period. In addition, there was no difference in the serum endotoxin levels between the two groups (S1 Figure).

### Distinct effect of periodontitis- and health-associated oral microbiota on the colonization of gut microbiota in germ-free mice

**Figure 2.**
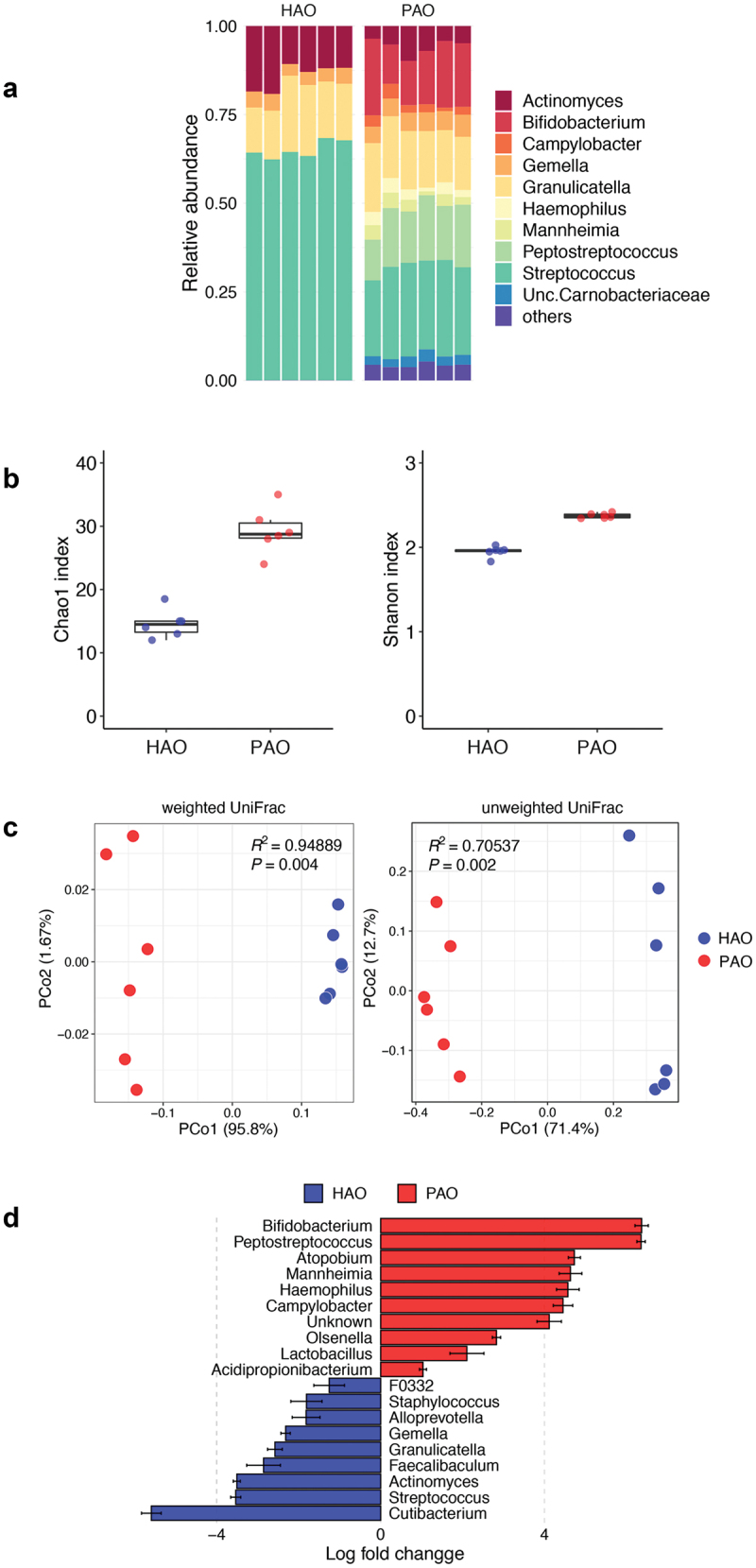
Comparative analysis of microbial composition in feces between periodontitis-associated oral microbiota-administered (PAO) and health-associated oral microbiota-administered (HAO) mice (n = 6/group). (**A**) Relative abundance of microbial groups at the genus level in the PAO and HAO mice. (**B**) Alpha diversity for Chao1 richness and Shannon diversity index of each experimental group. (**C**) Principal coordinate analysis score plot of the gut microbiota profiles of each experimental group using weighted and unweighted UniFrac distances. (**D**) Significantly different taxa as determined by ANCOM-BC at the genus level.

Despite significant differences in the phylum-level taxa of the administered oral microbiota, it seemed to be similar in microbial composition at the phylum level after colonization in the gut (S2 Figure). A total of 1,259,335 high-quality reads were detected in the fecal samples, with 52,472 ± 1,525 reads per sample. The difference in gut microbial composition between the two groups was clearly demonstrated by genus-level taxa and the analyses of α- and β-diversities. The microbial composition at the genus level differed between PAO and HAO mice (Figure 2A). Compared to HAO mice, both Chao1 and Shannon indices were significantly higher in PAO mice (*P* < 0.001), reflecting the higher α-diversity of administered saliva samples (Figure 2B). PCoAs based on the weighted and unweighted UniFrac distances clearly separated the two groups, and these samples were clustered and significantly segregated along the PC1 axis (Figure 2C). Differentially abundant taxa between PAO and HAO mice were analyzed using ANCOM-BC. *Bifidobacterium, Peptostreptococcus, Atopobium, Mannheimia, Campylobacter, Olsenella, Lactobacillus*, and *Acidpropionibacterium* were enriched in PAO mice; whereas *Cutibacterium, Streptococcus, Actinomyces, Faecalibaculum, Granulicatella, Gemella, Alloprevotella*, and *Staphylococcus* were enriched in HAO mice (Figure 2D).

## Periodontitis- and health-associated oral microbiota have a distinct effect on gene expressions in the liver

**Figure 3.**
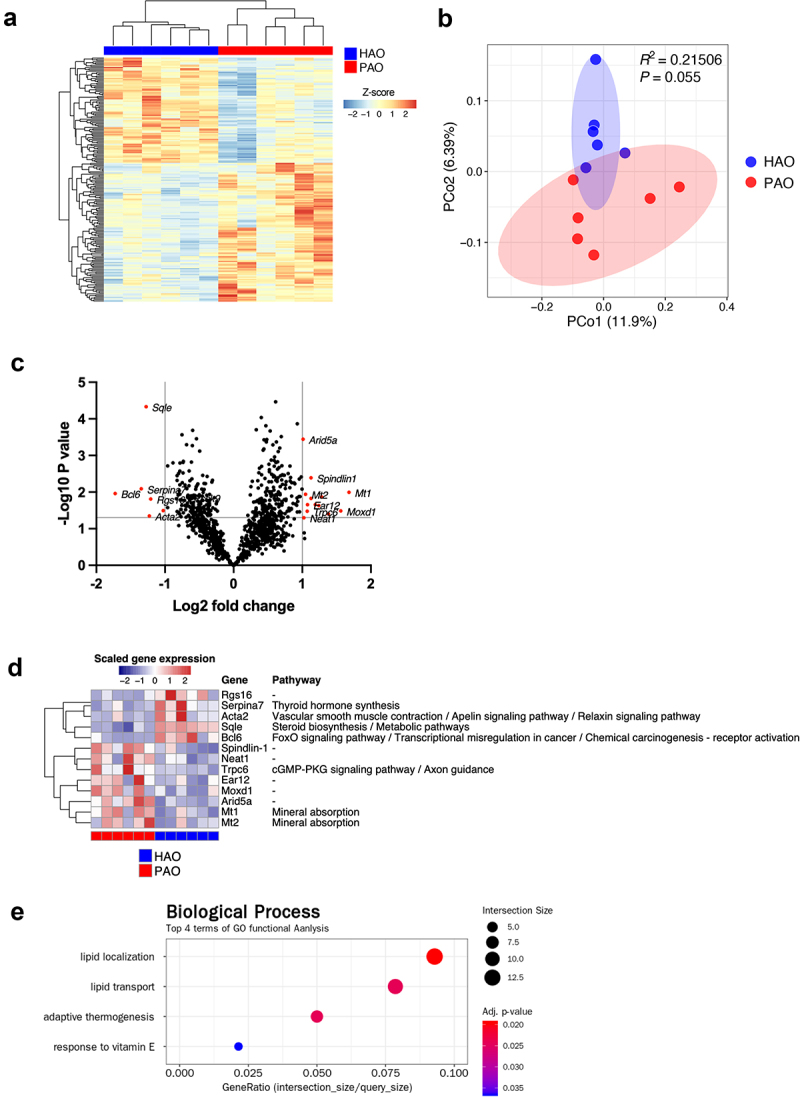
Gene expression profile in the liver of periodontitis-associated oral microbiota-administered (PAO) and health-associated oral microbiota-administered (HAO) mice (n = 6/group). (**A**) Hierarchical clustering heatmap of differentially expressed genes in the gut. Differentially expressed genes (DEGs) are listed along the Y-axis in the order that they clustered, as indicated by the colored line along the Y-axis. Each column contains expression values for an individual animal with groups indicated along the X-axis and clustering indicated by the dendrogram above the figure. (**B**) Principal coordinate analysis based on Bray-Curtis dissimilarity of the PAO and HAO mice gene expression profile. (**C**) Volcano plot of DEGs. The red dots represent up and down regulation according to the difference in expression (fold change of > 2) and significance (*P* < 0.05) in PAO mice compared with HAO mice. (**D**) Heatmap of the DEGs identified by volcano plot. (**E**) Gene set enrichment analysis of KEGG pathways. The size of the dots corresponds to the number of genes in the reference gene set. The color of the dots corresponds to the adjusted *P*-value.

The expression profiles of genes in the liver showed distinct patterns when compared between the two experimental groups, although individual variability was obvious (Figure 3A). PCoA identified the PAO and HAO mice as likely separated clusters (Figure 3B). Next, we aimed to identify those transcripts in the liver that were most significantly up or downregulated by the administration of periodontitis-associated or health-associated oral microbiota. As demonstrated in the volcano plot, *Spindlin1, Neat1, Trpc6, Ear12, Moxd1, Arida5a, Mt1*, and *Mt2* were significantly upregulated in PAO mice, whereas *Rgs16, Serpina7, Acta2, Sqle*, and *Bcl6* were significantly upregulated in HAO mice (Figure 3C). Although none of these genes showed statistically significant differentiation after adjustment using the Benjamini–Hochberg method, they were presented in a heatmap and assigned to their respective functions concerning the Kyoto Encyclopedia of Genes and Genomes (KEGG) pathways (Figure 3D). Gene enrichment and functional annotation analysis revealed that lipid localization, lipid transport, adaptive thermogenesis, and response to vitamin E were significantly enriched in GO terms (Figure 3E).

**Figure 4.**
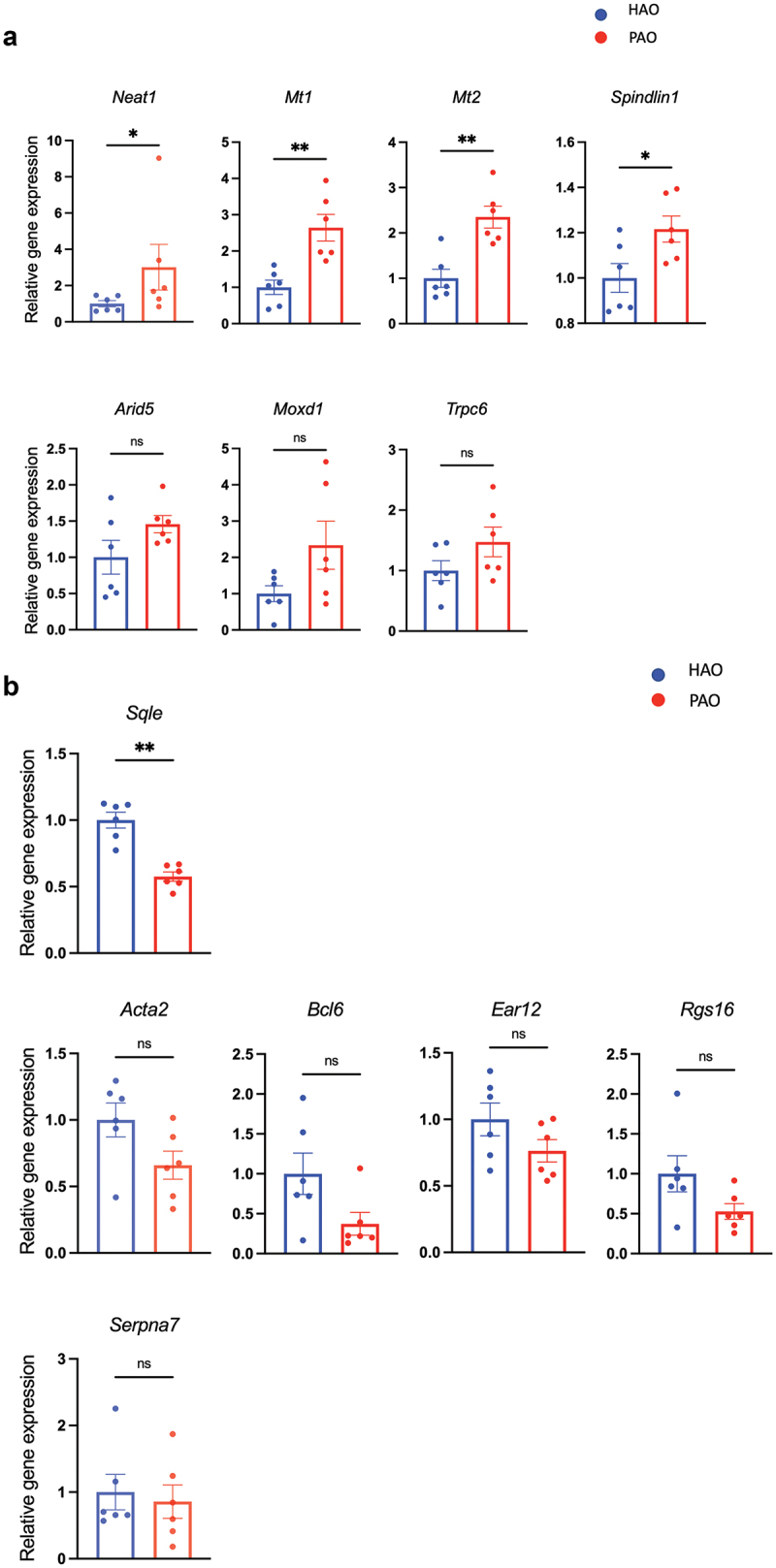
Relative mRNA expression of the differentially expressed genes (DEGs) by volcano plot analysis (n = 6/group). (**A**) Quantitative real-time polymerase chain reaction (qPCR) quantification of mRNA levels of genes expressed higher in the liver of periodontitis-associated oral microbiota-administered (PAO) mice. (**B**) qPCR quantification of mRNA levels of genes expressed higher in the liver of health-associated oral microbiota-administered (HAO) mice. Data are expressed as mean ± SEM; * *P* < 0.05, ** *P* < 0.005; Mann–Whitney U-test.

In line with the RNA-sequencing data, quantitative RT-PCR analyses of candidate genes characterizing each experimental group confirmed significantly elevated expression of *Neat1, Mt1, Mt2*, and *Spindlin1* (Figure 4A) and reduced expression of *Sqle* in the mice by oral gavage of the periodontitis-associated oral microbiota (Figure 4B). All of these transcripts are involved in lipid and glucose metabolism to some extent. *Bmal1, Dbp*, and NAFLD-related liver clock genes [21], also showed a significant difference between the two groups (S3 Figure).

## The composition of oral microbiota affects the serum metabolite profile

The only difference between the two experimental groups was in the microbial composition of the administered saliva samples. Therefore, if the serum metabolite profile of PAO mice is different from that of HAO mice, it could be solely attributable to the difference in colonized gut microbiota composition.

A PCA was conducted to obtain an overview of the differences between the groups. As shown in Figure 5A, the two experimental groups were clearly differentiated along the PC2 axis. Volcano plot analysis of differentially detected metabolites and subsequent pairwise analysis demonstrated that coumaric acid, 2-hydroxyisobutyric acid, and imidazolelactic acid levels were elevated; whereas the levels of ophthalmic acid, uric acid, and γ-Glutamylvalin (γ-Glu-Val) were decreased in PAO mice compared to HAO mice (Figures 5B and 5C).


**Figure 5.**
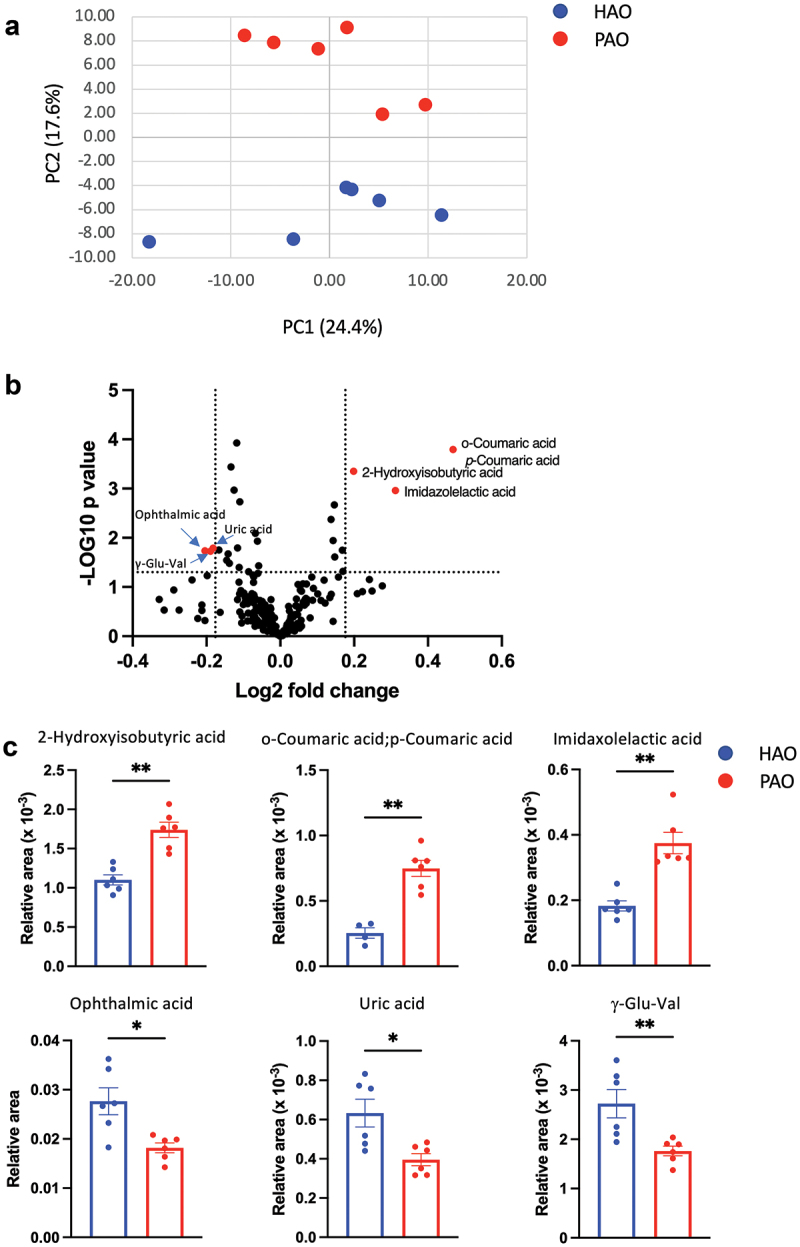
Oral gavage of periodontitis-associated oral microbiota and health-associated oral microbiota have distinct effects on serum metabolomic profile (n = 6/group). (**A**) Principal component analysis (PCA) of serum metabolites from periodontitis-associated oral microbiota-administered (PAO) and health-associated oral microbiota-administered (HAO) mice. (**B**) Volcano plot showing individual metabolites of PAO and HAO mice. Red plots represent significantly different metabolites (fold change of > 1.5 and *P* < 0.05). (**C**) Pairwise comparisons of significantly changed metabolites between PAO and HAO mice. Data were expressed as mean relative area ± SEM; * *P* < 0.05, ** *P* < 0.01, Mann–Whitney U-test. (**D**) Heatmap showing the correlation expression between 30 differential metabolites and the amplicon sequence variants. The correlation analyses were based on the Spearman correlation coefficient test. * *P* < 0.05, ** *P* < 0.01, *** *P* < 0.001.

**Figure 5.**
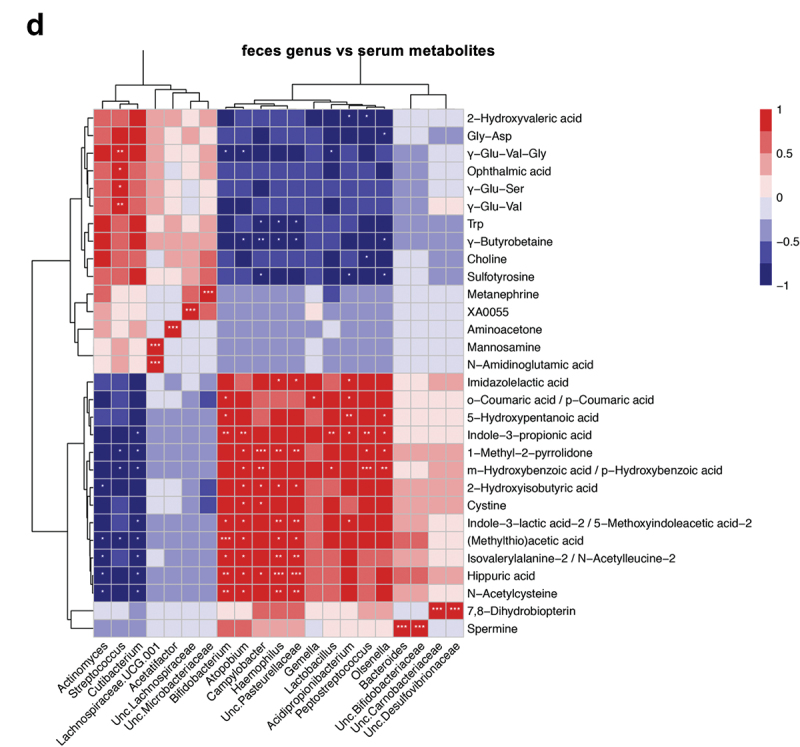
(continued).

We performed correlation analyses of the gut microbiota and serum metabolomic profiles due to the significant difference between PAO and HAO mice. The heatmap shows that the gut microbiota characteristics of PAO mice significantly correlate with some metabolites that have been reported to be associated with diseases such as 2-hydroxyisobutyric acid (2-HIBA), hydroxybenzoic acid, and indole-3-lactic acid. A strong correlation was observed for *Bifidobacterium, Atomobium, Campylobacter*, and *Haemophilus*. Conversely, *Streptococcus*, which was characteristic of HAO mice, significantly correlated with metabolites such as ophthalmic acid, uric acid, and γ-Glu-Val (Figure 5D).

## Discussion

We and others have shown that oral gavage of a single periodontopathic bacterium or ligature-induced periodontitis significantly impacts systemic conditions via the modulation of the gut ecology in mice [6–8,22–27]. However, the study conditions did not necessarily reflect the physiological state. In this study, human whole-salivary microbiota was administered to germ-free mice to make the experiment as physiological as possible. Moreover, although increasing evidence suggests that oral microbiota other than periodontopathic microbiota colonize the gut of otherwise healthy individuals [4], the impact of gut-colonizing oral microbiota on systemic health is largely unknown.

In this study, taxa with a high abundance in the saliva did not necessarily successfully colonize the gut. For example, *Bifidobacterium* with extremely low abundance in the saliva of both groups was highly enriched in PAO mice. Conversely, *Faecalibaculum* (previously known as *Faecalibacterium*) was highly enriched in HAO mice. *Haemophilus*, with a relatively high abundance in both groups, demonstrated a skewed presence in PAO mice. Furthermore, consistent with a previous study [4], periodontopathic microbiota, including red-complex bacteria, was barely detectable in PAO or HAO mice. These results suggest that special habitat selection, which may be derived from the composition of the salivary flora, is important.

The differences in colonized oral microbiota in the gut affected the hepatic gene expression profile. Correlation analysis demonstrated that the genes whose expressions were elevated in PAO mice were generally related to liver pathologies, such as steatosis, fibrosis, cancer, diabetes, and inflammation. *Neat1*, a long non-coding RNA, reportedly aggravates NAFLD by regulating the c-Jun/SREBP1c axis [28] and miR-212-5p/GLIA3 [29]. It also regulates fibrosis by targeting theβ-catenin/SOX9/TGF-β1 pathway [30]. Consistent with this observation, the expression of *Srebf1* tended to be higher in PAO mice than in HAO mice (S3 Figure). *Neat1* is also involved in liver cancer via activation of the Wnt/β-catenin pathway [31]. This pathway is also activated by *Spindlin1* and promotes cancer cell proliferation [32]. The Wnt signaling pathway is not only involved in tumor formation, but also in hepatic metabolism, suggesting its role in metabolic disorders such as diabetes [33]. *Mt1* and *Mt2*, which are considered to play pivotal roles in the metabolism of essential metals, also play a role in the prevention of oxidative and endoplasmic reticulum (ER) stress [34]. The elevation of *Mt1* and *Mt2* levels suggested that oxidative and/or ER stress responses occur in PAO mice. In support of this, ER stress-related genes (*Ddit4*) were significantly elevated in the livers of PAO mice compared to those in HAO mice. Therefore, the elevation of *Mt1* and *Mt2* is considered a compensatory response.

Nevertheless, the expression of only one gene, *Sqle*, which plays a key role in the synthesis of steroids, was significantly elevated in HAO mice compared to PAO mice. Reduced expression of *Sqle* has been reported in the liver of streptozotocin-induced diabetic mice [35] and is associated with the onset of diabetes [36]. Thus, ingestion of periodontitis-associated oral microbiota induces the alteration of gene expression in a disease-associated profile in the liver.

The metabolic profile of the serum was significantly different between PAO and HAO mice. This could be solely attributed to the difference in the gut microbiota composition. Furthermore, correlation analyses revealed significant correlations between the characteristic gut microbiota in PAO and HAO mice, either positive or negative, with particular metabolites. Consistent with the liver transcriptome analysis, health-associated oral microbiota (*Streptococcus*) was significantly associated with elevated levels of beneficial metabolites, such as γ-Glu-Val. γ-Glu-Val reportedly reduces gastrointestinal inflammation and TNF-α-induced vascular inflammation via the activation of calcium-sensing receptors.

Conversely, ophthalmic acid is considered a biomarker for oxidative stress [37], which increases with pathological changes in various organs, including the liver. It was higher in HAO mice than in PAO mice. However, whether the ophthalmic acid levels in HAO mice are due to elevated oxidative stress remains unknown. Moreover, the level of uric acid, a known risk factor for several diseases such as gout, was significantly higher in HAO mice than in PAO mice. Although uric acid expresses inflammatory and anti-inflammatory properties depending on its concentration [38], we did not determine the concentrations, and no correlation was observed with particular microbiota.

Among the characteristic metabolites in PAO mice, 2-HIBA is of particular interest because it is reportedly a biomarker for several metabolic diseases, such as type 2 diabetes [39] and adiposity [40]; as well as autoimmune diseases, such as rheumatoid arthritis [41]. Interestingly, there are significant associations between these diseases and periodontitis or periodontopathic microbiota, reinforcing the oral-gut connection as a causal mechanism connecting periodontitis and systemic disease. Coumaric acid is a polyphenol with bioactive properties, including antioxidant, anti-inflammatory, and lipid-lowering effects [42]. Elevated coumaric acid also suggested higher oxidative stress in PAO mice. Considering the gene expression profile and histology of the liver and glucose intolerance in PAO mice, a higher level of coumaric acid may be considered a compensatory response. Furthermore, some metabolites associated with colorectal cancer (hydroxybenzoic acid, indole-3-lactic acid-2/5-methoxyindoleacetic acid-2, and hippuric acid) were significantly positively correlated with the gut microbiota of PAO mice.

Thus, the multi-omics approach utilized in this study uncovered the role of the oral microbiota in the gut in systemic conditions. However, this study had several limitations. Experiments using germ-free mice are a good model for examining the colonization and disease phenotype of the human fecal microbiota. However, when examining the effects of the oral microbiota, conventional mice with their own adapted microbiota as an additional control are preferred. Saliva samples from a healthy individual and a periodontitis patient represent the typical microbial compositions of the corresponding clinical statuses. While differences in age have been reported to affect the bacterial microbiota [43], it has also been reported that there are no significant differences in the subgingival microbiota of younger and older patients with periodontitis [44]. However, given the high inter-individual variability of the microbiota, a sufficient number of donors or pooled material from several donors is a better way to compare the effects induced by microbiomes from periodontitis patients and healthy donors [45]. Further studies are needed to clarify the effect of the oral microbiota on the gut microbiome and their subsequent systemic effects.

## Supplementary Material

Supplemental Material

## Data sharing

The microbiome and transcriptome datasets presented in this study can be found in online repositories. The names of the repositories and accession numbers can be found below:

http://www.ncbi.nlm.nih.gov/bioproject/, PRJDB13217.

https://www.ncbi.nlm.nih.gov/, GSE198570.
