# Supplementary material for: Dysbiotic human oral microbiota alters systemic metabolism via modulation of gut microbiota in germ-free mice
Source: J Oral Microbiol. 2022 Aug 11;14(1):2110194. doi: 10.1080/20002297.2022.2110194 (PMC9373767; doi:10.1080/20002297.2022.2110194)
Supplement: Supplemental Material [file ZJOM_A_2110194_SM9280.zip › Supplementary files/Supple Tables R1_Clean copy.docx]

**Supplementary Tables**

**S1Table**

Demographic data of the donors

|  | Healthy subject | Periodontitis patient^*^ |
| --- | --- | --- |
| Age (years) | 35 | 63 |
| Gender | M | M |
| Smoking status | Non -smoker | Non-smoker |
| BMI | 24.3 | 20.3 |
| HbA1c (%) | 5.4 | 5.3 |
| Total cholesterol (mg/dL) | 174 | 162 |
| LDL-cholestrol (mg/dL) | 110 | 83 |
| HDL-cholestrol (mg/dL) | 52 | 69 |
| Triglyceride (mg/dL) | 77 | 58 |
| CRP (mg/dL) | 0.01 | 0.02 |
| Number of teeth | 24 | 26 |
| Plaque control record (%) | 2.8 | 52.9 |
| Mean PPD (mm) | 2.2 | 4.0 |
| Mean CAL (mm) | 2.2 | 5.1 |
| BOP (% positive sites) | 4.3 | 46.8 |

BMI: body math index, PPD: probing pocket depth, CAL: Clinical attachment level, BOP: bleeding on probing.

Plaque control record and BOP were calculated by dividing the number of positive sites by the total number of sites (6 sites/teeth) and expressed as percentages.

Mean PPD and CAL were calculated by sum of each measure (6 sites/teeth) was divided by the total number of sites.

*: Clinical diagnosis of periodontitis was based on tooth sites with PPD ≧ 6mm, CAL ≧ 5mm, and BOP > 30%.

**S2 Table**

Relative abundance (%) of salivary microbiota at genus level

| Genus | Health-associated saliva | Periodontitis-associated saliva |
| --- | --- | --- |
| *Streptococcus* | 0.43 | 0.20 |
| *Prevotella* | 0.034 | 0.054 |
| *Fusobacterium* | 0.028 | 0.067 |
| *Porphyromonas* | 0.052 | 0.055 |
| *Treponema* | 0 | 0.016 |
| *Aggregatibacter* | 0.0010 | 0.0090 |
| *Filifactor* | 0 | 0.013 |
| *Tannerella* | 0 | 0.0063 |
| *Bifidobacterium* | 0.0020 | 0 |

Relative abundance (%) of salivary microbiota at species level

| Species | Health-associated saliva | Periodontitis-associated saliva |
| --- | --- | --- |
| *Streptococcus mitis* | 0.27 | 0 |
| *Fusobacterium nucleatum* | 0 | 0.011 |
| *Prevotella intermedia* | 0 | 0.013 |
| *Porphyromonas gingivalis* | 0 | 0.033 |
| *Filifactor alocis* | 0 | 0.0094 |
| *Tannerella forsythia* | 0 | 0.0060 |
| *Treponema denticola* | 0 | 0.0016 |

**S3 Table**

Primers used for the quantitative real-time PCR

| Gene | Primer sequences | |
| --- | --- | --- |
|  | Forward | Reverse |
| *Mt1* | ACGCTGCGAATGGGTTTACG | ATCGCTGCTCTGGAGTTTACG |
| *Mt2* | CAAACCGATCTCTCGTCGAT | AGGAGCAGCAGCTTTTCTTG |
| *Spindlin1* | CCCCATTCGGGAAGACAC | ACAGGGAAGGATTCACAGG |
| *Arid5* | CAGCACCTCCGGCCAAA | CTTGAAGCCAAGATGGGGCA |
| *Moxd1* | TTCGTTGCAATTCCCTCCACAC | TGGGTTTTCTGCATTGCACCTG |
| *Trpc6* | ATCATTCTCGCTGCACATTGCC | TGGCTGCATTCTGTACACTTGC |
| *Sqle* | ATAAGAAATGCGGGGATGTCAC | ATATCCGAGAAGGCAGCGAAC |
| *Serpina7* | ACTCCACGAACCTACTCCTTCA | AGGCAATGGCAAGTGACTGT |
| *Rgs16* | AGGTTGTTTCCCTCTGCACTTG | TCGGCACAACACAACAGAACAC |
| *Bcl6* | TGCGCCATCCCTTTTTGAAGTG | ACGACAAGCATGACGCAGAATG |
| *Ear12* | TGGAAATCCAAGTGGCTTGTGC | TGTTCTTCTCCGACTGGTGATG |
| *Ddit4* | CTCTGGGATCGTTTCTCGTC | GACACCCCATCCAGGTATGA |
| *Srebf1* | GGAGCCATGGATTGCACATT | GGCCCGGGAAGTCACTGT |
| *Bmal1* | CCTAATTCTCAGGGCAGCAGAT | TCCAGTCTTGGCATCAATGAGT |
| *Dbp* | CACCGTGGAGGTGCTAATGA | ACTAGAGCGGGACAGTTCG |
| *Gapdh* | TCAACAGCAACTCCCACTCTT | ACCCTGTTGCTGTAGCCGTAT |
