# Supplementary material for: Dysbiotic human oral microbiota alters systemic metabolism via modulation of gut microbiota in germ-free mice
Source: J Oral Microbiol. 2022 Aug 11;14(1):2110194. doi: 10.1080/20002297.2022.2110194 (PMC9373767; doi:10.1080/20002297.2022.2110194)
Supplement: Supplemental Material [file ZJOM_A_2110194_SM9280.zip › Supplementary files/Supplementary legend.docx]

**Supplementary Figure 1**. Effect of periodontitis-associated oral microbiota and health-associated oral microbiota on the recipient mice. (**A**) Blood glucose levels at the indicated times after intraperitoneal load of glucose (1g/kg) and area under the blood concentration curve (AUC) in periodontitis-associated oral microbiota-administered (PAO; n=4) and health-associated oral microbiota-administered (HAO; n=3) mice. *P* < 0.05 PAO vs HAO; Unpaired t-test. (**B**) Changes in body weight during the experimental period, and liver weight and liver/body weight ratios of PAO and HAO mice (n=6/group). (**C**) Serum endotoxin levels in PAO and HAO mice (n=6/group). Data are expressed as the mean ± SEM. (**D**) Hematoxylin and eosin staining of the liver (scale bars, 100 μm) and epididymal adipose tissues of health-associated oral microbiota-administered (HAO) and periodontitis-associated oral microbiota-administered (PAO) mice. (**E**) F4/80 immunostaining of epididymal adipose tissues of HAO and PAO mice.

**Supplementary Figure 2.** Phylum level taxa of administered salivary microbiota (donor) and salivary microbiota composition of recipient mice.

**Supplementary Figure 3.** Relative mRNA expression of the non-alcoholic fatty liver disease (NAFLD)-associated genes in periodontitis-associated oral microbiota-administered (PAO) and health-associated oral microbiota-administered (HAO) mice (n=6/group).

Data are expressed as mean ± SEM; * *P* < 0.05; Mann–Whitney U-test.
